# Deep genetic structure at a small spatial scale in the endangered land snail *Xerocrassa montserratensis*

**DOI:** 10.1038/s41598-021-87741-7

**Published:** 2021-04-23

**Authors:** Cristina Català, Vicenç Bros, Xavier Castelltort, Xavier Santos, Marta Pascual

**Affiliations:** 1 Departament de Genètica, Microbiologia i Estadística and IRBio, Universitat de Barcelona, Diagonal 643, 08028 Barcelona, Spain; 2 Oficina Tècnica de Parcs Naturals, Diputació de Barcelona, Urgell 187, 08036 Barcelona, Spain; 3 ETSEA, Departament de Ciències del Sòl i Medi Ambient, Universitat de Lleida, Alcalde Rovira Roure, 191, 25198 Lleida, Spain; 4 CIBIO/InBIO, Centro de Investigação em Biodiversidade e Recursos Genéticos, Universidade do Porto, Campus Agrário de Vairão, R. Padre Armando Quintas s/n, 4485-661 Vairão, Portugal

**Keywords:** Biodiversity, Conservation biology, Evolutionary biology, Population genetics, Zoology

## Abstract

Species with small geographic ranges do not tend to have a high genetic structure, but some land snail species seem to be an exception. *Xerocrassa montserratensis*, an endangered land snail endemic to Catalonia (northeastern Iberian Peninsula), is an excellent model to study the processes affecting the phylogeography of specialized species of conservation concern. This species is restricted to xerophilous stony slopes and occurs within a small and fragmented area of ca. 500 km^2^. We sequenced the COI barcode region of 152 individuals from eight sites covering the entire range of the species. We found four genetic groups mostly coincident with their geographic distribution: a central ancestral group containing shared haplotypes among five localities and three groups restricted to a single locality each. Two of these derived groups were geographically and genetically isolated, while the third and most differentiated group was not geographically isolated. Geomorphologic and paleoclimatic processes during the Pleistocene can explain the divergence found between populations of this low dispersal species with historical fragmentation and secondary contacts. Nonetheless, recent passive large dispersal through streams was also detected in the central group. Overall, our study uncovered four evolutionary units, partially matching morphologically described subspecies, which should be considered in future conservation actions.

## Introduction

Invertebrate species represent the majority of multicellular organisms but are often neglected from conservation policies mostly due to lack of knowledge^1^. Land snails are highly diverse in the number of species although frequently unknown because of cryptic morphological speciation^2^. Projections since the 1980s estimate that 7% of land snails have been probably lost in front of the suggested 0.04% considering all taxa^3^. Although few genetic studies focused on European Mediterranean land snails, this group is composed by ca. 2700 species with a high rate of endemism and evolutionary diversification^4^. Land snails are organisms with very low dispersal abilities^5^. Some snail species have large distribution ranges, often associated to generalist ecological requirements^6–8^. However, many species have small distribution ranges and a considerable ecological specialization^9,10^. Life-history traits such as low mobility and ecological specialization make land snails good candidates to exhibit a high genetic structure^11^. Phylogeographic studies can provide information on genetic diversity and historical demographic processes such as isolation, gene flow and range expansion/contraction^12,13^. This approximation can help delineating conservation measures of threatened endemic species, such as some land snails^10,14^, and thus in setting species recovery priorities^15^.

Land snails of the genus *Xerocrassa* are distributed across the Mediterranean basin^16^, and include ca. 50 described species in the European side of the basin plus many subspecies^17^. Some species are morphologically cryptic and only molecular studies may uncover specific delimitations^14^. Many species are concentrated in Greece and in the Balearic Islands, and most of them are endemics (17 species in Greece and 11 in Balearic Islands), indicating the potential for genetic differentiation due to ecological specialization and low dispersal ability^10,18^. High endemism is also observed in the mainland, as exemplified by *Xerocrassa montserratensis*, a species restricted to the north-eastern part of the Iberian Peninsula with a small geographic distribution (< 3600 km^2^) and an area of occupancy of 448 km^2^^19^, fragmented in several isolated patches^20^. This snail shows a high habitat specialization, living almost exclusively in mountain xerophilous bare stony slopes of conglomerate lithology with narrow soil and little shrub and grass vegetation^21^. Currently, bare stony slopes are patchily distributed in isolated points surrounded by dense unsuitable forest and scrubland^22^.

At the end of the XIXth century three subspecies were described based on morphological traits of the shell: *X. m. montserrantesis*^23^*, X. m. betulonensis*^24^ and *X. m. delicatula*^25^. The distribution of these subspecies was limited to a few localities^25^. However, there is some controversy on their taxonomic status in the literature. One morphological study suggested that the subspecies *X. m. betulonensis* was a different species^26^. Nonetheless, a recent study that analysed characters of the shell and anatomical traits of the reproductive organs in individuals from the different areas failed to identify morphological differences in the traits historically used to discriminate among the three subspecies^20^.

Our study aims at understanding the processes structuring the populations of ecological specialist species with reduced distribution ranges by analysing the genetic structure of *Xerocrassa montserratensis* across its whole distribution range. Specifically, we (1) evaluate the genetic diversity of the species; (2) analyse the differentiation among populations; (3) test if the population genetic structure is explained by expansion, isolation by distance, or barriers to gene flow, and (4) investigate the validity of its subspecies. We hypothesize that the species will present high inter-population genetic differentiation caused by its suitable habitat fragmentation and low dispersal ability.

## Results

### Genetic diversity and population differentiation

**Figure 1:**
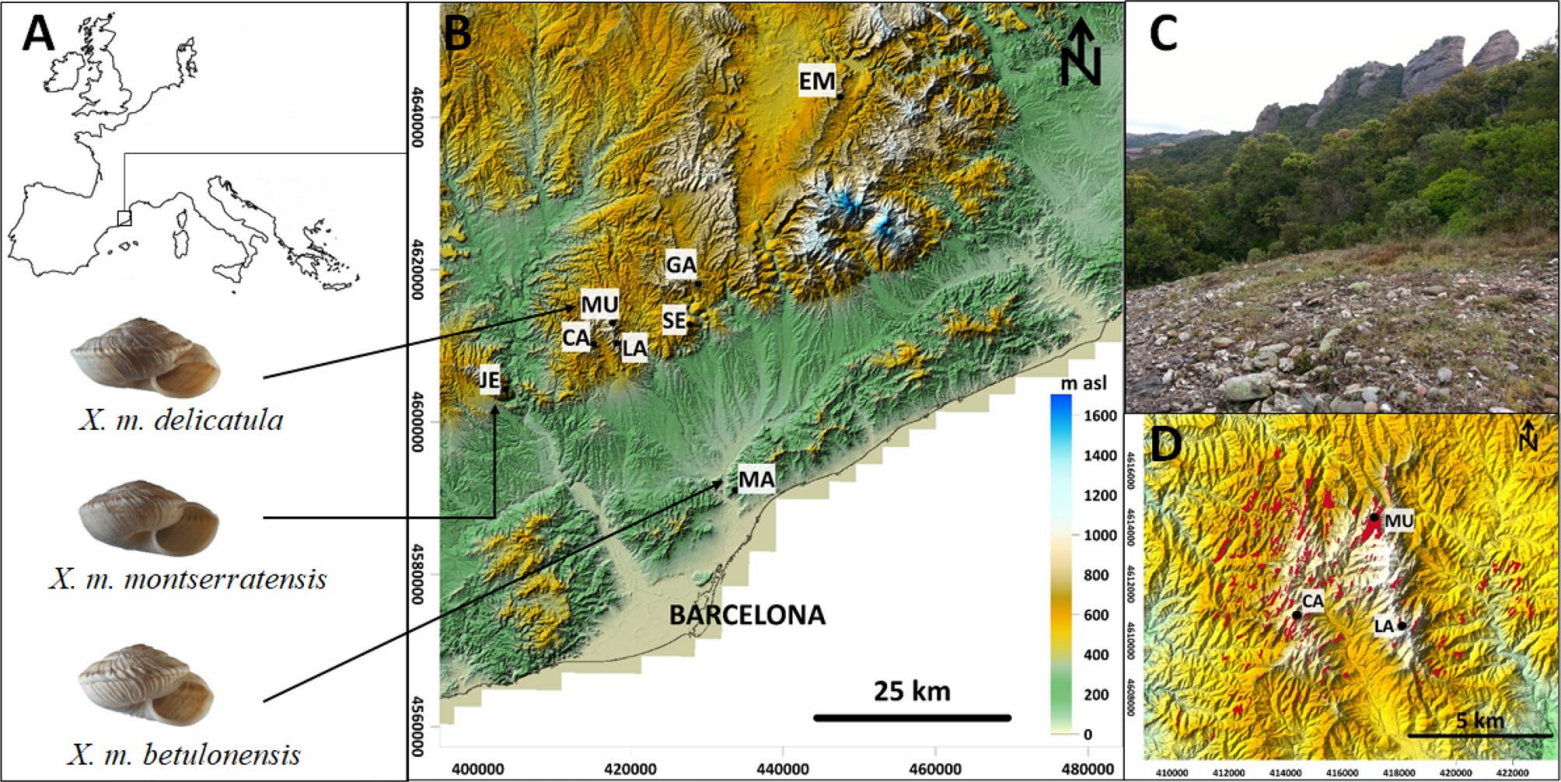
Distribution range, habitat and sampling information of *Xerocrassa montserratensis*. **(A)** Distribution map of *X. montserratensis* with shell photographs of the three morphologically described subspecies. The arrows point to their *locus typicus*^20^. **(B)** Location and codes of the eight sampling sites (Table 1). **(C)** Photography of a bare stony slope, the preferred habitat of *X. montserratensis.* **(D)** Distribution of bare stony slopes (red patches) in Sant Llorenç del Munt i l’Obac Natural Park including the three locations sampled in this park. The map in **(A)** was performed with ArcGIS 10x (ESRI, https://www.esri.com/). The maps in **(B,D)**, are in UTM coordinates for zone 31 T, and have been plotted with Surfer20 (Golden Software, https://www.goldensoftware.com/).

A total of 615 bp of the Cytochrome Oxidase I (COI) barcode fragment were aligned for 152 *Xerocrassa montserratensis* individuals from eight localities across the whole distribution range of the species (Fig. 1, Table S1). Overall, 33 different haplotypes were found (Table S2), of which 30 were private, meaning that they were found in only one locality. Total genetic diversity was high in both haplotype (0.91 ± 0.01) and nucleotide (0.011 ± 0.001) diversity. No differentiation in nucleotide diversity was found between localities (Table S3). However, haplotype diversity varied significantly between some of the localities (Table S3) with Montcau, Castellsapera and Els Munts showing higher haplotype diversity values (Table 1). Population diversity did not deviate from neutrality, except for Sant Jeroni (D = − 1.962, p = 0.01), although this deviation seems not due to a population expansion given that R_2_ values were not significant (Table 1).

**Table 1:** Genetic diversity values and neutrality tests of *Xerocrassa montserratensis* from the analysed localities.

Locality	Code	N	h	S	AR	Hd	π (%)	D	R_2_
Sant Jeroni	JE	21	5 (5)	10	2.8	0.486 ± 0.124	0.195 ± 0.108	− 1.962*	0.151
Montcau	MU	19	8 (8)	19	5.9	0.877 ± 0.044	0.626 ± 0.162	− 1.119	0.146
Castellsapera	CA	20	9 (8)	22	5.8	0.832 ± 0.063	0.649 ± 0.155	− 1.366	0.102
La Mola	LA	20	2 (1)	1	1.0	0.505 ± 0.056	0.082 ± 0.009	1.430	0.253
Gallifa	GA	13	3 (1)	2	2.0	0.410 ± 0.154	0.088 ± 0.036	− 0.462	0.164
Sentmenat	SE	20	4 (1)	3	2.5	0.647 ± 0.072	0.125 ± 0.022	− 0.244	0.133
Marina	MA	20	3 (1)	5	1.9	0.426 ± 0.122	0.167 ± 0.070	− 0.832	0.103
Els Munts	EM	19	5 (5)	5	3.6	0.731 ± 0.080	0.267 ± 0.032	0.450	0.160
Total	–	152	33	51	–	0.909 ± 0.014	1.131 ± 0.074	− 0.721	0.067

*N* number of individuals analysed, *h* number of haplotypes, private haplotypes in parentheses, *S* segregating sites, *AR* allelic richness for a sample of 12 individuals, *Hd* haplotype diversity ± standard deviation, *π* % of nucleotide diversity ± standard deviation; Tajima’s D and R_2_ neutrality tests.

*p values < 0.05.

**Figure 2:**
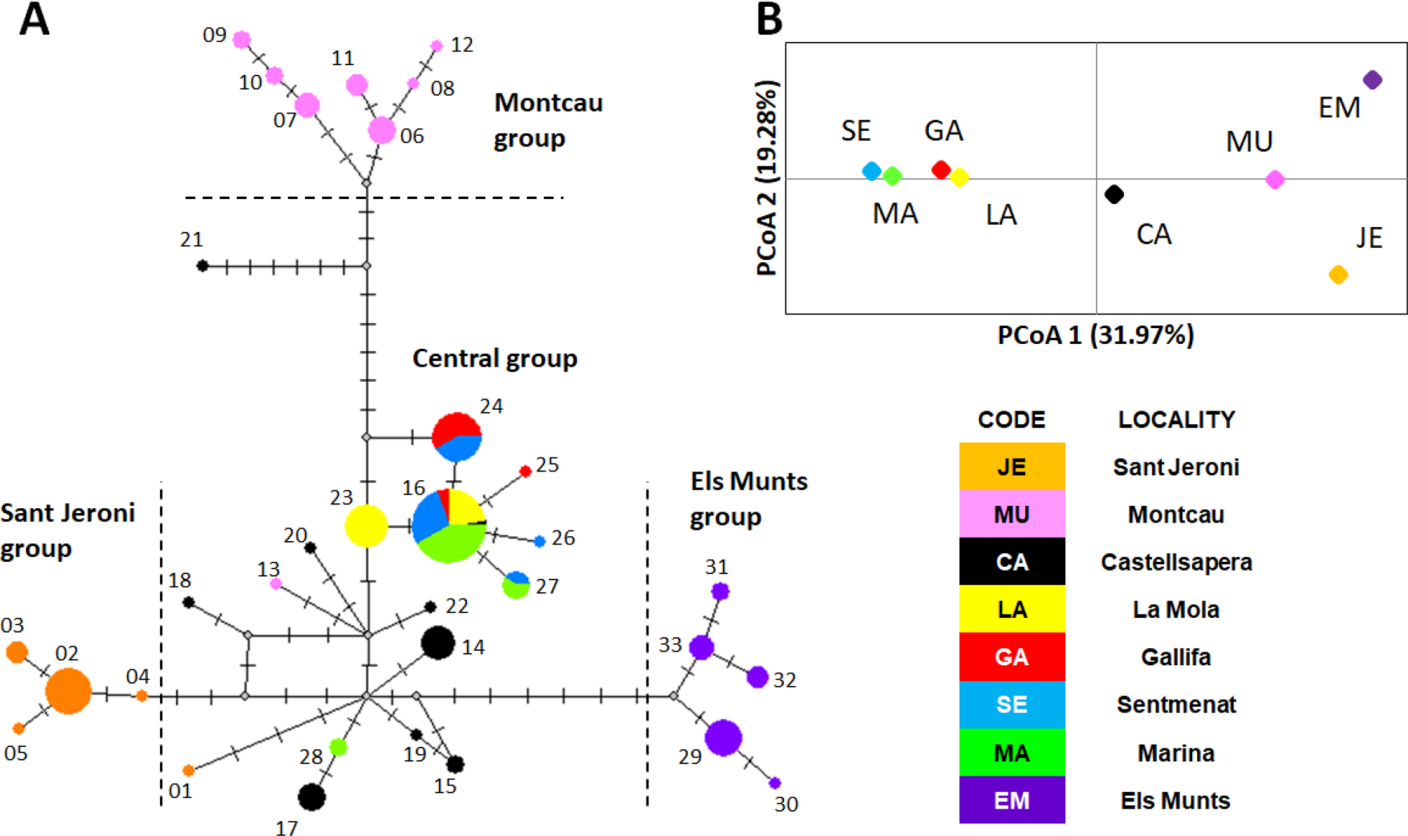
Median-joining network of *X. montserratensis* COI haplotypes with the four identified groups **(A)**. Each haplotype is identified with its number and circle colour coded by locality. The size of the circles is proportional to the number of sequences with the same haplotype. Dashes represent nucleotide changes between haplotypes. Principal Coordinates Analysis (PCoA) based on pairwise populations F_ST_ values **(B)**. The haplotype network was built and edited with Network 10 (https://fluxus-engineering.com/). The PCoA plot was built with GenAlEx (https://biology-assets.anu.edu.au/GenAlEx/).

Four well-differentiated groups were identified with the haplotype network (Fig. 2). A central group including haplotypes mostly present in five localities, and three peripheral groups, each almost restricted to a single locality. Sant Jeroni group was composed by 4 haplotypes exclusively found in this locality, in the Montserrat Mountain (Figs. 1 and S1), from where the species was described^23^. The Montcau group contained 7 haplotypes all from that locality, from where the subspecies *X. m. delicatula* was initially described (Fig. 1)^25^, and one highly differentiated haplotype from the neighbouring locality of Castellsapera. Finally, the Els Munts group was formed by 5 haplotypes only present in that geographically distant locality (Fig. 2). Interestingly, the Central group contained haplotypes from Marina, the only sampling site located near the coast. This group included individuals from the populations described as *X. m. betulonensis* (Marina, Gallifa and Sentmenat)^25^. In the case of Montcau and Sant Jeroni, it is interesting to emphasize that we also found haplotypes clustering in the central group (Fig. 2).

The differentiation among populations explained the largest variation (77.5%) of the global variance, as revealed by an AMOVA without any a priori grouping. The rest of the total variation (22.5%) was explained by differences within populations. In both cases, the differences were significant (P < 0.001). All pairwise populations’ comparisons (F_ST_ values) were significant after FDR correction except for the comparison between Sentmenat and Gallifa (Table 2). The plot representing the localities with a Principal Coordinates Analysis (PCoA) based on the pairwise F_ST_ matrix explained 51.25% of the differences between populations with the first two axes (Fig. 2). Marina (MA), Sentmenat (SE), Gallifa (GA) and La Mola (LA) were relatively close to each other, all of them with haplotypes in the central group of the network. Montcau (MU), Sant Jeroni (JE) and Els Munts (EM) were far away from the rest while Castellsapera (CA) had an intermediate ordination in the PCoA plot.

**Table 2:** Pairwise genetic distances between *Xerocrassa montserratensis* localities.

	JE	MU	CA	LA	GA	SE	MA	EM
JE		< 0.001	< 0.001	< 0.001	< 0.001	< 0.001	< 0.001	< 0.001
MU	0.834		< 0.001	< 0.001	< 0.001	< 0.001	< 0.001	< 0.001
CA	0.624	0.685		< 0.001	< 0.001	< 0.001	< 0.001	< 0.001
LA	0.871	0.789	0.427		< 0.001	< 0.001	< 0.001	< 0.001
GA	0.865	0.758	0.525	0.641		0.019	< 0.001	< 0.001
SE	0.849	0.785	0.515	0.421	0.182		0.004	< 0.001
MA	0.823	0.781	0.429	0.257	0.420	0.120		< 0.001
EM	0.892	0.855	0.722	0.891	0.893	0.889	0.872	

F_ST_ values are shown below the diagonal and P values above. P values < 0.013 are significant according to FDR correction. Locality codes as in Table 1.

No isolation by distance was observed with a Mantel Test when all localities were used (r = 0.439, P = 0.092; Figure S2), suggesting that genetic distances between populations were not explained by geographic distances. We suspected that some localities could be acting as outliers, according to the haplotype network and PCoA plot (Fig. 2). For instance, Montcau located in the same natural park as Castellsapera and La Mola (Fig. 1), and thus in close proximity to them, was highly differentiated. On the contrary, Marina although geographically distant had haplotypes in the central group and shared with other localities. For this reason, we performed Mantel Tests without these localities resulting in a significant isolation by distance when excluding either Montcau (r = 0.599, P = 0.048), Marina (r = 596, P = 0.009) or both (r = 0.745, P = 0.001) (Figure S2). Finally, we identified two barriers with the Monmonier Maximum Difference Algorithm: the first separated Els Munts from the rest, and the second separated Montcau. However, when using ‘pseudoslopes’ to reflect the change in genetic composition relative to the change in physical distance, a maximum differentiated peak was obtained around Montcau (Figure S3).

### Phylogenetic analysis and molecular dating

**Figure 3:**
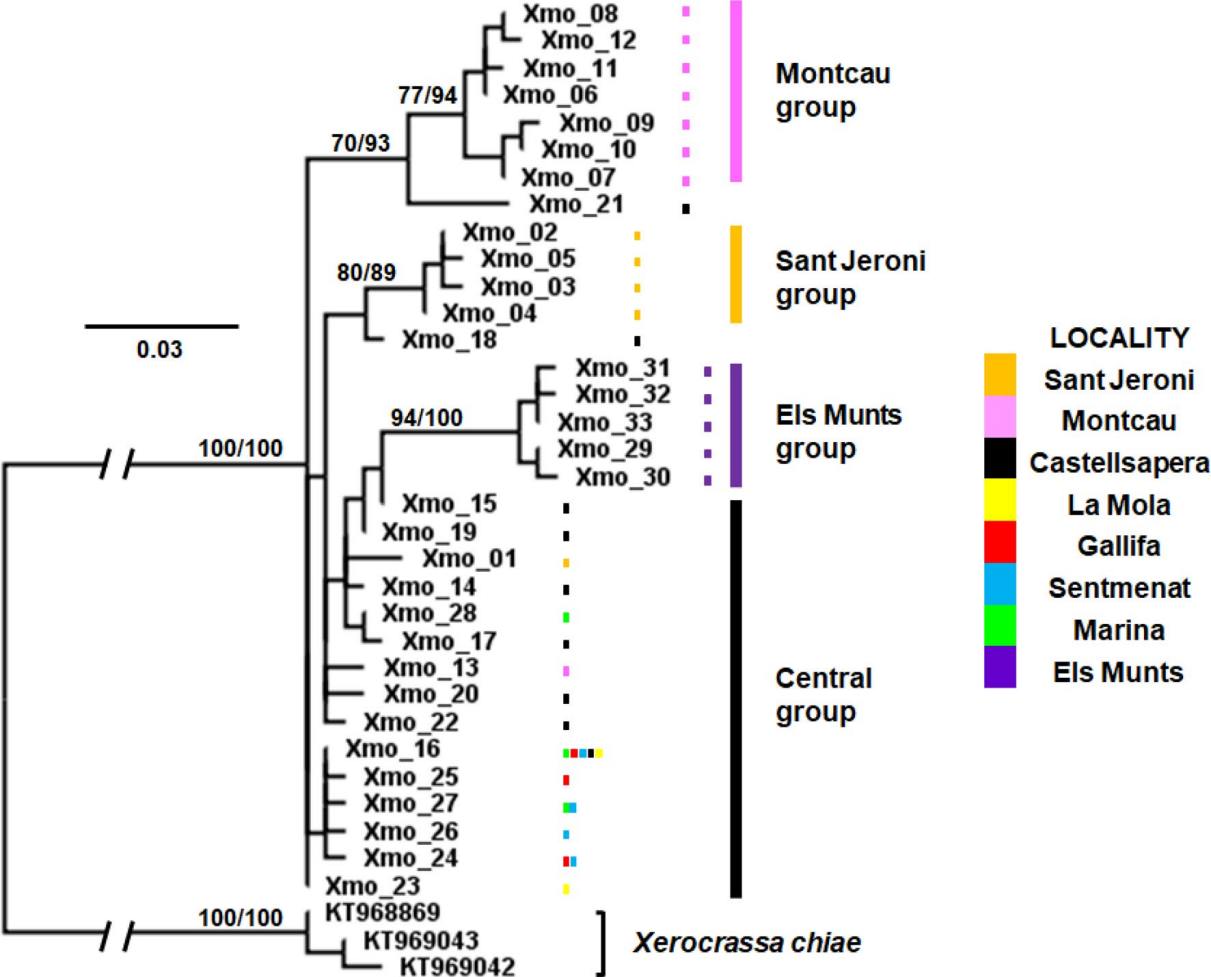
Maximum likelihood phylogenetic tree of *Xerocrassa montserratensis* COI haplotypes using sequences of *X. chiae* as outgroup (Table S4). Values at the nodes are only shown for high bootstrap values/posterior probabilities. The colours identify the locality where each haplotype has been detected. The groups are the same identified in the haplotype network in Fig. 2. The tree was edited using Figtree v. 1.4.4. (http://tree.bio.ed.ac.uk/software/figtree/).

**Figure 4:**
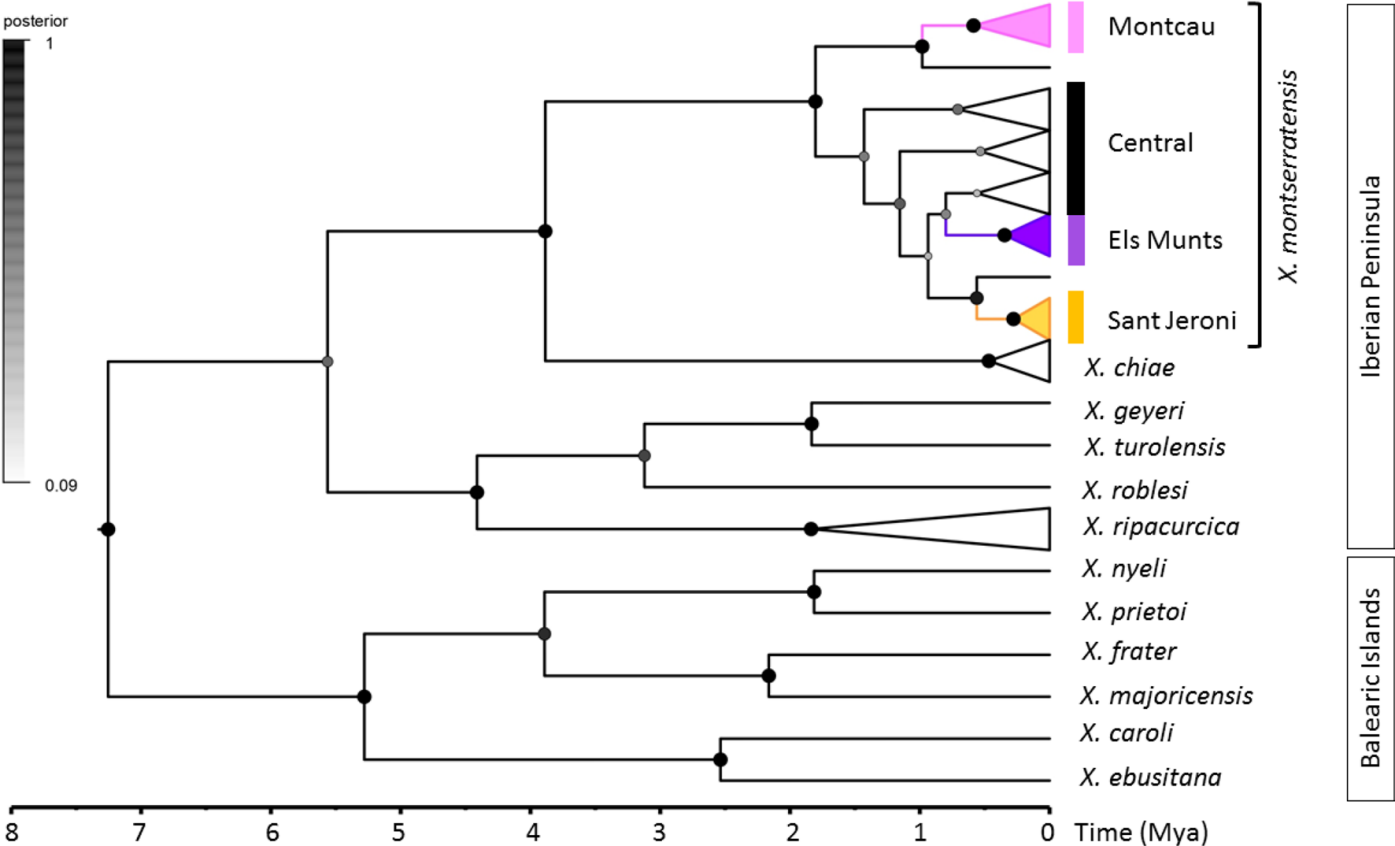
Calibrated Bayesian COI tree using BEAST, with the four groups observed for *Xerocrassa montserratensis*, colour coded as in the haplotype network and phylogenetic tree (Figs. 2 and 3), and species of the Iberian Peninsula and the Balearic Islands (Accession numbers in Table S4). The divergence among species of the Balearic Islands was used to calibrate the tree. The dots in the nodes provide posterior probabilities with size and grading according to the scale bar. The big black dots represent a posterior probability > 0.99. The tree was edited using Figtree v. 1.4.4. (http://tree.bio.ed.ac.uk/software/figtree/).

The *X. montserratensis* haplotype phylogenetic trees obtained by Maximum likelihood (ML) and Bayesian inference (BI), using *X. chiae* as outgroup, were concordant and grouped all haplotypes with high support values (Fig. 3). The haplotypes in the central group of the network (Fig. 2) had a more basal position in the phylogenetic tree and presented low support values, while the three external groups in the network (Montcau, Sant Jeroni and Els Munts) had high support values with both phylogenetic methodologies (Fig. 3). Considering the differentiation in these three groups we carried out a phylogenetic reconstruction and molecular dating with BEAST to estimate the time of coalescence of the haplotypes in the different groups. We used as outgroups 11 species (Table S4), six from the three main clades of the Balearic Islands and five considered as the closest relatives of *X. montserratensis* from the Iberian Peninsula^18^. We based the separation of the three main clades of *Xerocrassa* species in the Balearic Islands around 5.3 ± 0.3 Mya, as done by previous authors^18^. The mean substitution rate per site and Myr was 0.0203 (95% HPD interval: 0.0136, 0.0274), within the range of the rate calculated for other land snails^27^. All *X. montserratensis* haplotypes grouped with high support values (Fig. 4) and the time to their most recent common ancestor was dated around (mean ± sd) 1.91 ± 0.64 Mya. We also estimated the coalescence time of the haplotypes in each of the three divergent groups, according to the network and the phylogenetic tree reconstruction. The most recent common ancestor of all haplotypes found in the Montcau group, exclusive from that locality, dated from 0.65 ± 0.31 Mya (Fig. 4), from the Els Munts group dated from 0.40 ± 0.23 Mya, and from Sant Jeroni, coalesced 0.33 ± 0.21 Mya.

## Discussion

In this work, we assessed the population structure of the xerophilous land snail *Xerocrassa montserratensis*, an endemic species of a small region in Catalonia (northeastern Iberian Peninsula)*.* We detected significant genetic differentiation among localities as expected for a species with a low mobility and a high habitat specialization. The population differentiation followed an isolation by distance model with some exceptions. On one hand, the two closest neighbouring localities showed a high differentiation. On the other hand, one of the most geographically distant localities showed a low differentiation. Finally, three of the main mitochondrial genetic groups encountered matched the distribution area of subspecies morphologically described in the XIXth century but the taxonomy of which had recently been discussed controversially.

### Population genetic diversity and differentiation

Haplotype diversity was high in *Xerocrassa montserratensis* populations, as found in other terrestrial-snails^13,27,28^. This high genetic diversity could be explained by cryptic initial speciation processes, fragmentation of the suitable habitat, secondary contacts, local adaptation and ultimately reduced gene flow^14,29^. We found differences in genetic diversity among localities that could be related to their extent of suitable habitats, as observed in other species where a positive correlation between allelic richness and habitat patch size had been reported^30^. This pattern seems to be concordant in Sant Llorenç del Munt i l’Obac Natural Park (Fig. 1), where Montcau and Castellsapera, the two localities with the highest haplotype diversities, have the largest extent of bare stony slopes, whereas La Mola has only a small extension of suitable habitat, in agreement with the lowest values of haplotype diversity. In the study area bare stony slopes are patchily distributed and surrounded by oak forests that natural reforestation and fire can contract and expand in a dynamic process^22^. Thus, fluctuations in habitat extension impacting on population sizes in this species may determine their present diversity and differentiation. A similar pattern has been observed in *Xerocrassa* species from Crete Island due to gene flow barriers and population expansions^14^.

Although in our study most genetic differentiation among localities could be explained by an isolation by distance model, two localities deviated from this pattern: Marina and Montcau. In the case of Marina, the locality presented low genetic distance but high geographic distance from the central populations of *X. montserratensis*. This low genetic differentiation could be due to a recent colonization event, most probably through passive dispersal. Despite their low mobility, passive dispersal has been documented for some land snails due to anthropogenic activities^5,31^. Dispersal capacity has been shown to be negatively correlated with body size, and long distance passive dispersal also mediated through wildlife, water and wind^32–34^. Thus, *X. montserratensis* might be passively dispersed probably due to its small size. Based on morphological similarities of the shell, Bofill hypothesized that the snails from Marina derived from the populations of Gallifa and Sentmenat by passive dispersal through the Ripoll and Besòs Rivers^25^. These two localities are upstream of these rivers whereas Marina is located downstream of the Besòs River. Thus, more than 100 years later we confirmed Bofill’s hypothesis with molecular markers. The presence of *X. montserratensis* fossils found in Rubí^25^, 20 km downstream from its present distribution in Sant Llorenç del Munt i l’Obac Natural Park, suggests that passive dispersal along streams has occurred multiple times. This ability to disperse passively over large distances combined with the presence of suitable habitat may explain the current patchy distribution of this vulnerable species.

Conversely to Marina, Montcau showed high genetic differentiation with Castellsapera and La Mola populations, located less than 4 km apart, all of them within the Sant Llorenç del Munt i l’Obac Natural Park. High genetic differentiation at small spatial scale may be explained by a past fragmentation and posterior secondary contact, as already proposed in other land snail studies^12,29,35^. For instance, high genetic distances among nearby populations found in *X. mesostena* from Crete island were explained by geographic barriers and population expansion facilitated by deforestation^14^. Other studies have found high genetic differentiation in land snail species with a reduced geographic range^30,36^. All these examples suggest that a high genetic distance between nearby snail populations is a common pattern although the geographic and historical context may change among species.

### A paleogeographic scenario to explain *X. montserratensis* differentiation

In *X. montserratensis*, the time to the most recent common ancestor of all COI haplotypes was dated around 1.9 Mya, and the three peripheral haplotype groups, exclusive from a different locality, showed coalescent times between 0.3 and 0.6 Mya, placing the fragmentation of these populations into the Pleistocene. During this geologic period, glaciations have been proposed as mechanisms of population fragmentation in land snails, with interglacial periods promoting expansion from different refugia due to the contraction of ice sheets or changes in vegetation cover^14,36^. The coalescent time of the three peripheral haplotype groups of *X. montserratensis* match different glacial periods^37,38^. Thus, changes in vegetation cover during the Pleistocene climatic oscillations, due to fire and reforestation, could explain the present biogeographic haplotype distribution in *X. montserratensis*, since this species is currently observed in non-forested and recently burned areas^22,39^.

Alternatively, geomorphological processes, such as a reorganization of the water drainage system, could also be responsible for paleogeographic population isolations, explaining the present genetic structure of *X. montserratensis*. Jointing and homoclinal shifting (i.e. changes in the position of homoclinal ridges in a down-dip direction) are known to shape drainage rearrangements^40^. In the study area, homoclinal shifting occurred thanks to the rifting along transversal basement faults (Vallès-Penedès and Amer Faults, Figure S1). For instance, in the Guilleries area (Figure S1), homoclinal shifting has caused a scarp retreat of nearly 20 km to the west^41^, which might contribute to the genetic isolation of the population of Els Munts. At Sant Llorenç and Montserrat areas, a set of joints oriented SSW–NNE occurred, exerting significant control on initial drainage rearrangement. In this area we have calculated a scarp retreat of ca. 5 km due to homoclinal shifting^41^, which may have isolated snail populations from La Mola and Montcau, genetically very distinct despite being geographically close.

In addition, species with small dispersal distances and low population sizes might be prone to show phylogeographic breaks that can arise without any barrier to gene flow, especially at maternally inherited markers^42^. Thus further research at the genome wide level is needed to evaluate the drivers of the mitochondrial differentiation identified between the neighbouring localities of *X. montserratensis* within the Sant Llorenç del Munt i l’Obac Natural Park.

### Mitochondrial revalidation of *X. montserratensis* taxonomy

The subspecies described more than 100 years ago are concordant with our observed mitochondrial analyses. Thus, the nominal form *X. m. montserratensis* would correspond to the Sant Jeroni molecular group coinciding with the locality where the species was described^23^. The subspecies described as *X. m. betulonensis*^24^ matches some of the locations in the central molecular group and thus should not be considered a different species as previously claimed based on morphological characters^26^, and seems to be the subspecies with the largest distribution area. The subspecies *X. m. delicatula*^25^ could correspond to the Montcau molecular group, from where the subspecies was described. This group is the most genetically differentiated, although the presence of haplotypes from the central group in the localities of Montcau would suggest incomplete lineage sorting or present gene flow through secondary contact. Future studies with genome wide markers using the same individuals are necessary to discriminate between these two scenarios. Finally, the differentiated molecular group found in Els Munts, the geographically most distant population, located at the Guilleries (Catalan Transversal Range), could also be considered a different subspecies with slightly morphological differences since individuals in this locality have more rounded shell and a less marked carinate shell border^20^. Despite morphological variation in the shell shape and size among different *X. montserratensis* populations, Martinez-Ortí & Bros^20^ recently stated that morpho–anatomical characters from both the shell and reproductive system cannot be used to discriminate the described taxa. Morpho-static evolution has been described in some snail species arising from molecular but non-ecological differentiation^43^. Moreover, discrepancies between nuclear, mitochondrial and morphological data can result from initial stages of the speciation processes with ongoing gene flow^44,45^. Thus, the subspecies status in *X. montserratensis* should be considered preliminary since only mitochondrial data has been used. Additional studies using genome-wide markers should be undertaken to evaluate whether parapatric *X. montserratensis* populations have current gene flow, further providing the potential to uncover adaptation processes^46,47^.

### Conservation implications

Our study provides new information for conservation management actions to be considered by policymakers and stakeholders at the Natural Parks, where most of *X. montserratensis* populations are located. The genetically differentiated groups found in this species could be considered different evolutionary significant units, matching the initially described morphological subspecies, with the identification of a potential new subspecies. According to IUCN, *X. montserratensis* is listed as endangered because its reduced distribution range and habitat specialization. Moreover, its populations are decreasing since a low number of specimens have been found in some populations (https://www.iucnredlist.org/species/22254/9368348 accessed December 2020)^19^. The fact that we have found high genetic structuring implies that there is not only the need of species protection but also to protect each genetic group in a coordinated manner since the risk of extinction is higher. Although the species inhabits protected Natural Parks, conservation plans are mostly based on larger-sized fauna and flora. Our study helps to put more emphasis in this kind of fauna (no arthropod invertebrates), which normally are not considered flag species and deserve less interest from conservation institutions, and highlights the role of genetic studies in setting species priorities in conservation management plans.

## Material and methods

### Distribution range and field sampling

*Xerocrassa montserratensis* is a land snail species of less than 1.5 cm of shell diameter^20^ inhabiting bare stony slopes of conglomerate lithology in the northeastern Iberian Peninsula (Fig. 1). This conglomerate lithology is distributed in a clastic sedimentary belt that extends from the Montserrat area to the Guilleries area, bordering the western margin of the Montseny Massif at heights between 800 and 1200 m (Figure S1 and Table S1). The land snail *X. montserratensis* is listed as Endangered in the IUCN Red List of Threatened Species^19^.

At the end of the XIXth century three subspecies were described based on morphological traits of the shell (Fig. 1): *X. m. montserrantesis*^23^*, X. m. betulonensis*^24^ and *X. m. delicatula*^25^. The subspecies *X. m. delicatula* was only described in the area of La Mata at Sant Llorenç del Munt i l’Obac Natural Park. On the other hand, *X. m. betulonensis* was only found in three localities, Gallifa and Sentmenat (Catalan Prelitoral Range) and Marina (Catalan Litoral Range). Finally, *X. m. montserrantesis* was described from individuals collected in the Montserrat Mountain, where the species was initially described^23^. In a recent morphologic study, a more globose shell shape and less carinated shell periphery was described in individuals from Els Munts^20^.

Snails were collected from eight locations, most of them in Natural Park reserves, covering the known range of the species including the localities where the different subspecies were described and with previous morphologic analyses (Fig. 1 and Table S1). All the collection sites are located across the Catalan Prelitoral Range with the exceptions of Els Munts located in the Catalan Transversal Range and Marina located in the Catalan Litoral Range. The lithology of all these localities is composed by xerophilous conglomerates and highly fragmented bare stony slopes, except Marina which is characterized by Paleozoic granodiorites and located at the lowest altitude.

A total of 152 snail individuals were collected between autumn 2013 and autumn 2014, except for La Mola which was sampled in winter 2015. For conservation management of this endangered species, juveniles were prioritized over adults for collection. Juveniles are more abundant in the localities and have lower survival capacity during the summer drought experienced in Mediterranean environments. The permit to collect the specimens was granted by the corresponding authorities of the natural parks managed by Diputació de Barcelona, Patronat de la Muntanya de Montserrat, and Servei de Fauna i Flora of the Generalitat de Catalunya. Approximately 20 samples were taken from each locality and preserved in absolute ethanol for further genetic analyses (Table 1). The epiphragm was broken to ensure correct material preservation for DNA extraction. Two specimens of *X. ripacurcica* from Congost de Montrebei (Lleida) were collected and sequenced in the present work.

### DNA extraction, amplification and sequencing

For each specimen, the shell was broken and a small fragment of foot tissue was cut and dried to remove ethanol. Total genomic DNA was extracted using QIAamp DNA Mini Kit (QIAGEN), according to the manufacturer’s protocol, and resuspended in 50 µl AE buffer. The universal primers LCO1490/HCO2198^48^ were used to amplify the barcode region of the Cytochrome Oxidase I (COI) gene. PCR amplifications were carried out in a total volume of 20 µl including: 2 µl of 5 × Buffer (GoTaq, Promega), 1 µl of 25 nmol MgCl_2_, 0.5 µl of dNTP (1 mM), 0.4 µl of each primer (10 µM), 0.2 µl of Taq polymerase corresponding to 1 unit (GoTaq, Promega) and 1 µl of DNA. The PCR started with an initial denaturation at 94 °C for 5 min, followed by 35 cycles of a denaturation step at 94 °C for 1 min, an annealing step at 50 °C for 1 min and an elongation step at 72 °C for 1 min 30 s, and a final elongation step at 72 °C for 7 min. The amplified DNA was purified with Exo-SAP (0.2 U/µl Exonuclease and 0.2 U/µl Shrimp Phosphatase) at a proportion of 1:2 (ExoSap:PCR product) and the forward strand was sequenced by Macrogen or Scientific and Technologic Services at the Universitat de Barcelona. Sequence chromatograms were visually checked, aligned and cut to the same length with MEGA X^49^. We only used the forward primer because the obtained sequences had clear nucleotide peaks. Doubtful sequences were repeated for amplification and sequenced from both primers for reliability.

### Genetic diversity, haplotype network and population structure

Haplotype diversity, nucleotide diversity and their standard deviations were calculated for each population using DnaSP 6^50^. To evaluate differences in diversity between localities we carried out a permutation test with 10,000 replicates using genetic_diversity_diffs v1.0.6 (https://github.com/laninsky/genetic_diversity_diffs) ^51^. To compare the number of haplotypes among localities with different number of analysed individuals we calculated allelic richness with Contrib^52^. To identify signs of population demographic events deviating from neutrality we computed Tajima’s D neutrality test and R_2_ test of demographic expansion using DnaSP 6 for each locality separately and the whole area combined. Haplotype frequencies per locality, pairwise genetic distances (F_ST_) between localities and its significance, and the amount of variation found within and among localities (AMOVA) were calculated using Arlequin ver. 3.5.2^53^. The Benjamini-Yekutieli False Discovery Rate (FDR) correction^54^ was applied to account for multiple comparisons. The genetic relations among populations were visualized in a bi-dimensional plot by a principal coordinates analysis (PCoA) with GenAlEx^55^ using pairwise F_ST_ values. Correlations between pairwise population genetic (F_ST_) and geographic distance matrices were evaluated by Mantel tests and its significance assessed with 999 permutations using GenAlEx. Pairwise geographic distances were computed as the Euclidean distance in km between each two collecting sites. Finally, barriers to gene flow among localities were calculated with Alleles In Space^56^ using Monmonier Maximum Difference Algorithm and Interpolate Genetic Landscape Shape. Raw distances and the option “pseudoslope”, which corrects the genetic distances with the geographic ones, were also used to identify the barriers.

To show the number of nucleotide changes among haplotypes and their relationships we built a haplotype network using the Median Joining Network Algorithm with the software Network 10 (https://fluxus-engineering.com/).

### Phylogenetic analyses and molecular dating

Phylogenetic relationships among haplotypes were estimated using both Maximum Likelihood (ML) and Bayesian Inference (BI) methods. *Xerocrassa chiae* was used as outgroup, since it is the closest relative^18^. For phylogenetic reconstruction we used the HKY + G + I evolution model, as identified by the Bayesian information criteria in MEGA X^49^. For ML analysis, PhyML v 3.1^57^ was ran with 1000 replicates to obtain the bootstrap support values. MrBayes v. 3.2^58^ was used for BI analysis. The Bayesian posterior probabilities were obtained running 3 heated chains and 1 cold chain for two separate runs, with 1 × 10^7^ generations each, saving one tree every 1000 generations. A consensus tree was obtained after discarding the first 25% iterations as burn-in.

The time of divergence between groups of *X. montserratensis* was estimated using a Bayesian approach implemented in BEAST 1.10^59^. The analysis was based on the geographic calibration followed by Chueca et al.^18^ considering that the three main clades of *Xerocrassa* species within the Balearic Island diverged during the Messinian Salinity Crisis, 5.3 ± 0.3 Mya. Overall, we used 11 additional species (Table S4), six from the three main clades of the Balearic Islands and five considered the closest relatives of *X. montserratensis* from the Iberian Peninsula^18^. Two specimens of *X. ripacurcica* analysed in the present work were also included. We tested for substitution saturation in DAMBE 7^60^ and no saturation was obtained when considering all sites or each codon position separately. The Yule model was selected as a speciation model, and an exponential relaxed clock without correlation was used. Two independent runs were performed for 1 × 10^8^ generations, with a sampling every 10,000 generations. The two runs were combined using LogCombiner 1.10^59^ and the first 10% of trees for each run removed as burn-in. The parameters were verified with Tracer 1.7^61^ ensuring ESS > 200 for all parameters estimated. The highest credibility tree was identified with TreeAnnotator 1.10^59^ that summarizes all retained trees into a single consensus. Phylogenetic trees were visualized and edited using Figtree v. 1.4.4. (http://tree.bio.ed.ac.uk/software/figtree/).

## Supplementary Information

Supplementary Information.

## Data Availability

*Xerocrassa montserratensis* and *X. ripacurcica* haplotype sequences are deposited in GenBank (Accession numbers MW642508-40 and MW642546-7, respectively).
