# Supplementary material for: Deep genetic structure at a small spatial scale in the endangered land snail Xerocrassa montserratensis
Source: Sci Rep. 2021 Apr 23;11:8855. doi: 10.1038/s41598-021-87741-7 (PMC8065133; doi:10.1038/s41598-021-87741-7)
Supplement: Supplementary file 1 — Supplementary Information. [file 41598_2021_87741_MOESM1_ESM.pdf]

Deep genetic structure at a small spatial scale in the endangered land snail *Xerocrassa montserratensis*

Cristina Català, Vicenç Bros, Xavier Castelltort, Xavier Santos, Marta Pascual

SUPPLEMENTARY MATERIAL

Locality	CODE	N	UTM X	UTM Y	Elevation(m)
Sant Jeroni	JE	21	401531	4606441	1,100
Montcau	MU	19	416872	4613931	1,000
Castellsapera	CA	20	414472	4611027	910
La Mola	LA	20	418149	4610745	1,050
Gallifa	GA	13	427103	4617354	950
Sentmenat	SE	20	426815	4610916	600
Marina	MA	20	435006	4590980	125
Els Munts	EM	19	448011	4643259	848

Table S1. Information of *Xerocrassa montserratensis* sampled locations and number of analysed individuals (N). The UTM coordinates are in zone 31T.

	JE	MU	CA	LA	GA	SE	MA	EM
**Xmo_01**	1							
**Xmo_02**	15							
**Xmo_03**	3							
**Xmo_04**	1							
**Xmo_05**	1							
**Xmo_06**		5						
**Xmo_07**		4						
**Xmo_08**		1						
**Xmo_09**		2						
**Xmo_10**		2						
**Xmo_11**		3						
**Xmo_12**		1						
**Xmo_13**		1						
**Xmo_14**			7					
**Xmo_15**			2					
**Xmo_16**			1	8	2	10	15	
**Xmo_17**			5					
**Xmo_18**			1					
**Xmo_19**			1					
**Xmo_20**			1					
**Xmo_21**			1					
**Xmo_22**			1					
**Xmo_23**				12				
**Xmo_24**					10	7		
**Xmo_25**					1			
**Xmo_26**						1		
**Xmo_27**						2	3	
**Xmo_28**							2	
**Xmo_29**								9
**Xmo_30**								1
**Xmo_31**								2
**Xmo_32**								3
**Xmo_33**								4
**Total**	**21**	**19**	**20**	**20**	**13**	**20**	**20**	**19**

Table S2. Absolute frequencies of the 33 haplotypes of *X. montserratensis* found in each locality. Shared haplotypes are highlighted in grey. Population codes as in Table S1.

	**JE**	**MU**	**CA**	**LA**	**GA**	**SE**	**MA**	**EM**
**JE**		0.0043	0.0045	0.0011	0.0011	0.0007	0.0003	0.0007
**MU**	**0.3915**		0.0002	0.0054	0.0054	0.0050	0.0046	0.0036
**CA**	**0.3459**	0.0456		0.0057	0.0056	0.0052	0.0048	0.0038
**LA**	0.0195	**0.3719**	**0.3263**		0.0001	0.0004	0.0008	0.0018
**GA**	0.0755	**0.4669**	**0.4213**	0.0950		0.0004	0.0008	0.0018
**SE**	0.1617	**0.2298**	**0.1842**	0.1421	**0.2371**		0.0004	0.0014
**MA**	0.0594	**0.4509**	**0.4053**	0.0789	0.0161	**0.2211**		0.0010
**EM**	**0.2453**	0.1462	0.1006	**0.2257**	**0.3207**	0.0836	**0.3047**	

Table S3. Differences between localities on haplotype diversity (below the diagonal) and nucleotide diversity (above the diagonal). Differences in diversity were assessed by a permutation test with 10,000 replicates using genetic_diversity_diffs v1.0.6[1]. In bold significant values after FDR correction ($p<0.013$). Population codes as in Table S1.

Species	Locality	Province	Accession number	Reference
X. montserratensis	Sant Jeroni	Barcelona	MW642508	Xmo_01, present study
X. montserratensis	Sant Jeroni	Barcelona	MW642509	Xmo_02, present study
X. montserratensis	Sant Jeroni	Barcelona	MW642510	Xmo_03, present study
X. montserratensis	Sant Jeroni	Barcelona	MW642511	Xmo_04, present study
X. montserratensis	Sant Jeroni	Barcelona	MW642512	Xmo_05, present study
X. montserratensis	Montcau	Barcelona	MW642513	Xmo_06, present study
X. montserratensis	Montcau	Barcelona	MW642514	Xmo_07, present study
X. montserratensis	Montcau	Barcelona	MW642515	Xmo_08, present study
X. montserratensis	Montcau	Barcelona	MW642516	Xmo_09, present study
X. montserratensis	Montcau	Barcelona	MW642517	Xmo_10, present study
X. montserratensis	Montcau	Barcelona	MW642518	Xmo_11, present study
X. montserratensis	Montcau	Barcelona	MW642519	Xmo_12, present study
X. montserratensis	Montcau	Barcelona	MW642520	Xmo_13, present study
X. montserratensis	Castellsapera	Barcelona	MW642521	Xmo_14, present study
X. montserratensis	Castellsapera	Barcelona	MW642522	Xmo_15, present study
X. montserratensis	Castellsapera, La Mola, Gallifa, Sentmenat, Marina	Barcelona	MW642523	Xmo_16, present study
X. montserratensis	Castellsapera	Barcelona	MW642524	Xmo_17, present study
X. montserratensis	Castellsapera	Barcelona	MW642525	Xmo_18, present study
X. montserratensis	Castellsapera	Barcelona	MW642526	Xmo_19, present study
X. montserratensis	Castellsapera	Barcelona	MW642527	Xmo_20, present study
X. montserratensis	Castellsapera	Barcelona	MW642528	Xmo_21, present study
X. montserratensis	Castellsapera	Barcelona	MW642529	Xmo_22, present study
X. montserratensis	La Mola	Barcelona	MW642530	Xmo_23, present study
X. montserratensis	Gallifa, Sentmenat	Barcelona	MW642531	Xmo_24, present study
X. montserratensis	Gallifa	Barcelona	MW642532	Xmo_25, present study
X. montserratensis	Sentmenat	Barcelona	MW642533	Xmo_26, present study
X. montserratensis	Sentmenat, Marina	Barcelona	MW642534	Xmo_27, present study
X. montserratensis	Marina	Barcelona	MW642535	Xmo_28, present study
X. montserratensis	Els Munts	Barcelona	MW642536	Xmo_29, present study
X. montserratensis	Els Munts	Barcelona	MW642537	Xmo_30, present study
X. montserratensis	Els Munts	Barcelona	MW642538	Xmo_31, present study
X. montserratensis	Els Munts	Barcelona	MW642539	Xmo_32, present study
X. montserratensis	Els Munts	Barcelona	MW642540	Xmo_33, present study
X. chiae	Roquesblanques	Girona	KT968869	Chueca et al. 2017[2]
X. chiae	Bagà	Barcelona	KT969042	Chueca et al. 2017[2]
X. chiae	Bagà	Barcelona	KT969043	Chueca et al. 2017[2]
X. ripacurcica	Congost de Montrebei	Lleida	MW642546	Xri_01, present study
X. ripacurcica	Congost de Montrebei	Lleida	MW642547	Xri_02, present study
X. ripacurcica montsicciana	Congost de Ventamillo	Huesca	KT969057	Chueca et al. 2017[2]
X. ripacurcica ripacurcica	Circo de Armeña	Huesca	KT969041	Chueca et al. 2017[2]

X. ripacurcica ripacurcica	Cerler	Huesca	KT969052	Chueca et al. 2017[2]
X. ripacurcica ripacurcica	Sopeira	Huesca	KT969065	Chueca et al. 2017[2]
X. roblesi	Nàquera	Valencia	KT969055	Chueca et al. 2017[2]
X. geyeri	Penyagolosa	Castellón	KT969054	Chueca et al. 2017[2]
X. turolensis	Puerto de Villaroya	Teruel	KT969059	Chueca et al. 2017[2]
X. ebusitana	Cap de Barbaria	Formentera	KT969064	Chueca et al. 2017[2]
X. caroli caroli	Els Penjats	Ibiza	KT968896	Chueca et al. 2017[2]
X. frater frater	Montdragó Natural Park	Mallorca	KT969060	Chueca et al. 2017[2]
X. majoricensis	Son Vida	Mallorca	KT968950	Chueca et al. 2017[2]
X. nyeli	Cala d'Algariens	Menorca	KT969009	Chueca et al. 2017[2]
X. prietoi prietoi	Son Serra de Marina	Mallorca	KT969032	Chueca et al. 2017[2]

Table S4. Species, locality information and GenBank Accession numbers of the COI sequences used in the phylogenetic analyses in the present work and publication reference. The name of the haplotypes found in the present study are provided and their localities are separated by commas.

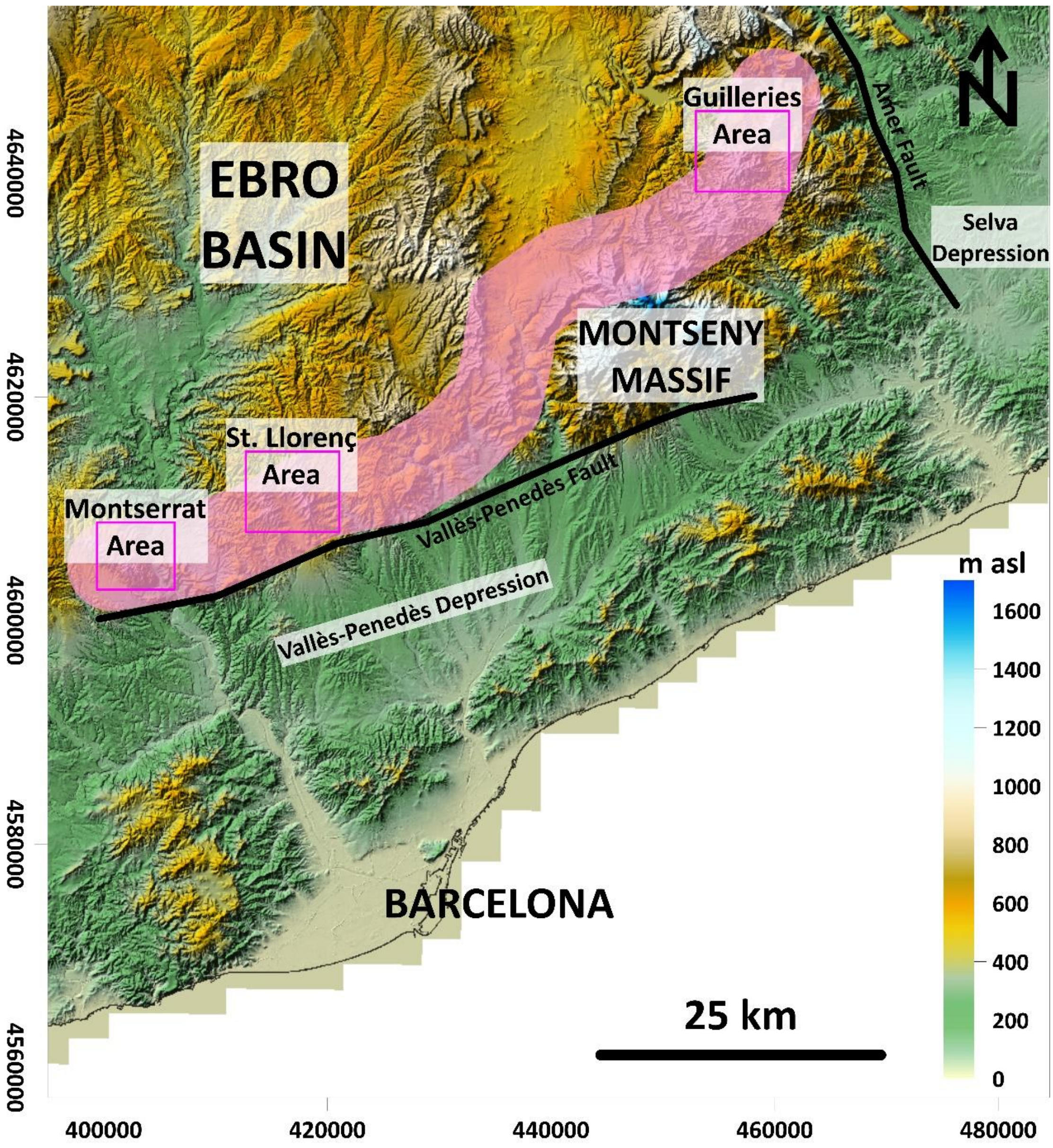

Figure S1. Siliciclastic sediments from the former Eocene coast outcrop in the south-eastern margin of the Ebro Basin. Such materials were deposited by the coastal alluvial fans of Montserrat and Sant Llorenç del Munt [3]. Coarse sandstones and microconglomerates outcrop in the eastern margin of the Ebro Basin, corresponding to marine platform siliciclastic deposits from the Folgueroles Sandstones Formation [4,5]. The two lithological units lie with lateral continuity forming a clastic sedimentary belt (in pink) that extended from the Montserrat area to the Guilleries area, bordering the western margin of the Montseny Massif. Characteristically, the clastic belt, at heights between 800 and 1,200 m asl, was the early specialized habitat of *Xerocrassa montserratensis*. The map, in UTM coordinates for zone 31T, has been plotted with Surfer20 (Golden Software).

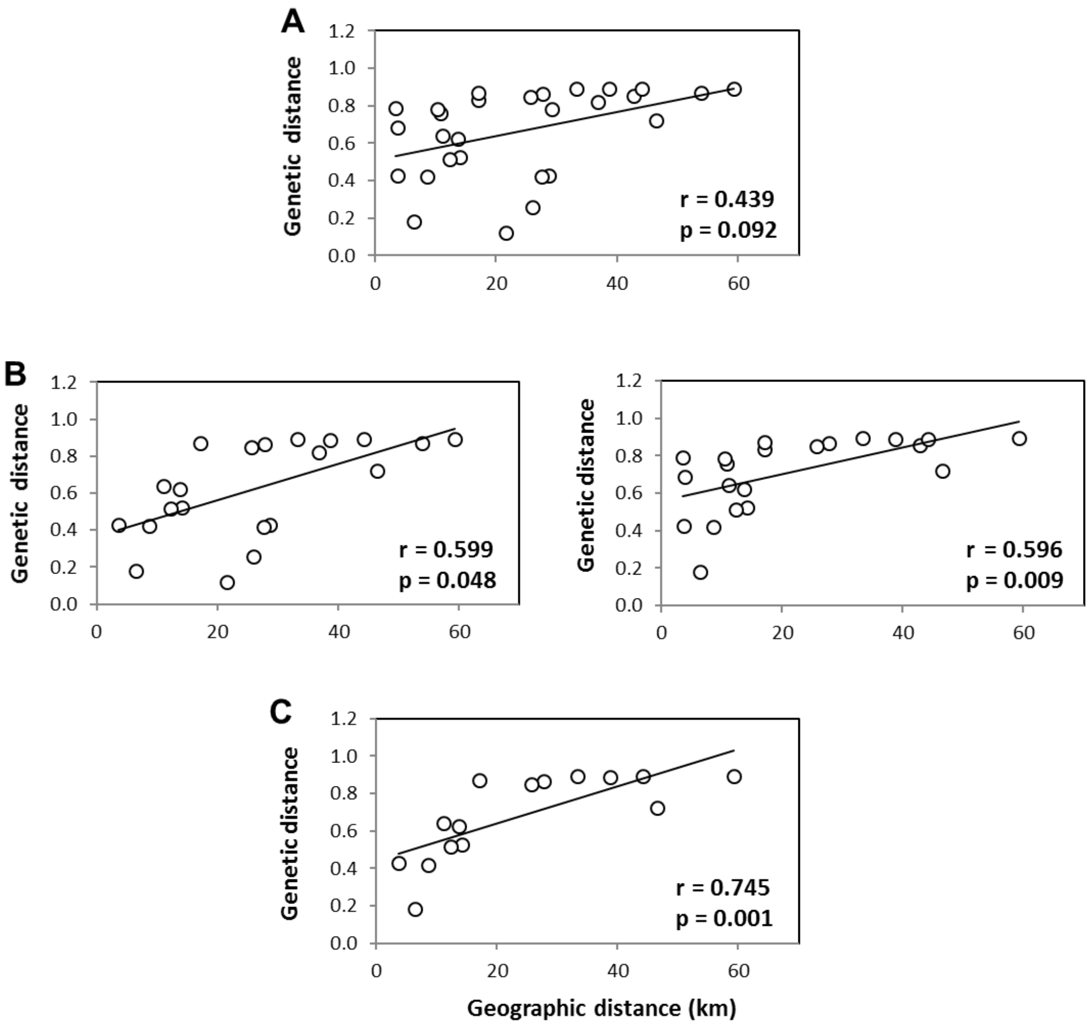

Figure S2. Mantel Tests showing the correlation between pairwise genetic (F_{ST}) and geographic (km) distances. (A) For all localities, (B) excluding MU (on the left), and MA (on the right), (C) excluding both MU and MA.

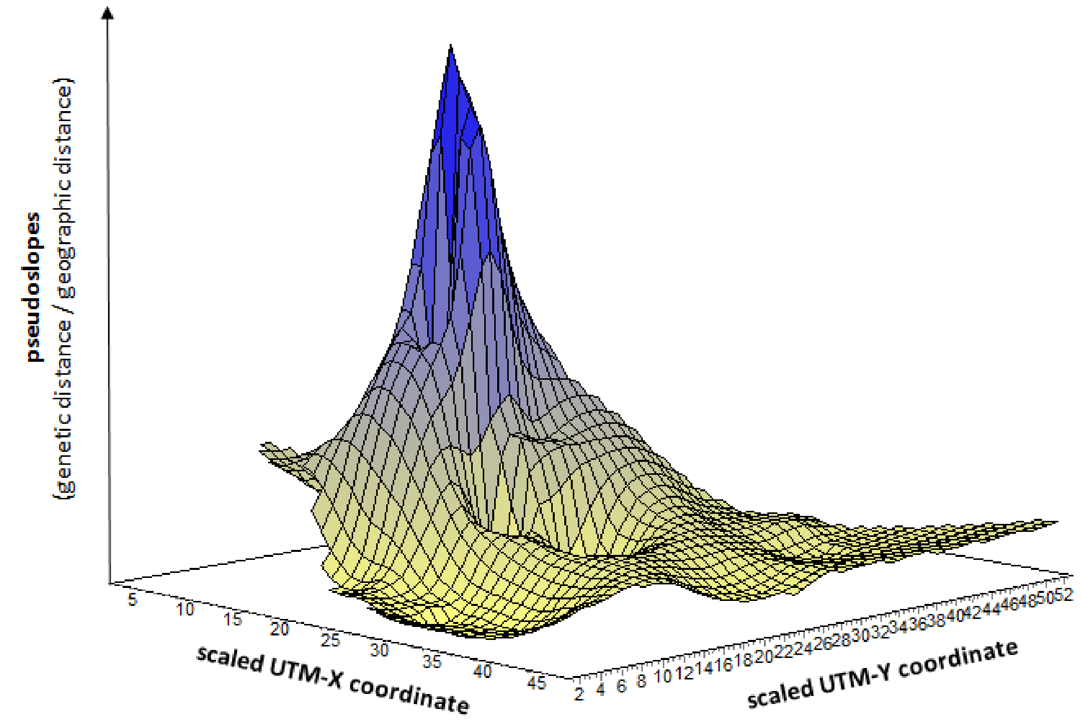

Figure S3. Spatial patterns of genetic differentiation using interpolation techniques [6]. Colours represent pseudoslope values. The blue peak indicates high levels of genetic differentiation at small spatial scales around Montcau.

References

1. Alexander, A. *et al.* What influences the worldwide genetic structure of sperm whales (*Physeter macrocephalus*)? *Mol. Ecol.* **25**, 2754–2772 (2016).

2. Chueca, L. J., Gómez-Moliner, B. J., Forés, M. & Madeira, M. J. Biogeography and radiation of the land snail genus *Xerocrassa* (Geomitridae) in the Balearic Islands. *J. Biogeogr.* **44**, 760–772 (2017).

3. López-Blanco, M. Estratigrafía secuencial de sistemas deltaicos en cuencas de antepaís: ejemplos de Sant Llorenç del Munt, Montserrat y Roda (Paleógeno, cuenca de antepaís surpirenaica). (PhD Thesis, Universitat de Barcelona, 1996).

4. Santisteban, C. & Taberner, C. Geometry, structure and geodynamics of a sand wave complex in the southeast margin of the Eocene Catalan Basin, Spain. in *Tide-influenced sedimentary environments and facies* (eds. De Boer, P. L., Van Gelder, A. & Nio, S. D.) 123–138 (D. Reidel Publishing Company, 1988).

5. Taberner, C., Dinarès-Turell, J., Giménez, J. & Docherty, C. Basin infill architecture and evolution from magnetostratigraphic cross-basin correlations in the southeastern Pyrenean foreland basin. *Bull. Geol. Soc. Am.* **111**, 1155–1174 (1999).

6. Miller, M. P. Alleles In Space (AIS): computer software for the joint analysis of interindividual spatial and genetic information. *J. Hered.* **96**, 722–724 (2005).
